# The genomic configurations driving antimicrobial resistance and virulence in colistin resistant *Pseudomonas aeruginosa* from an Egyptian Tertiary Oncology Hospital

**DOI:** 10.1371/journal.pgph.0004976

**Published:** 2025-08-05

**Authors:** Asmaa AbdulHak, Hamdallah H. Zedan, Hadir A. El-Mahallawy, Ahmed A. Sayed, Hend O. Mohamed, Mai M. Zafer

**Affiliations:** 1 Department of Microbiology and Immunology, Faculty of Pharmacy, Ahram Canadian University, Giza, Egypt; 2 Department of Microbiology and Immunology, Faculty of Pharmacy, Cairo University, Cairo, Egypt; 3 Department of Clinical Pathology, National Cancer Institute, Cairo University, Cairo, Egypt; 4 Genomics Program, Children’s Cancer Hospital Egypt, Cairo, Egypt; 5 Department of Biochemistry, Faculty of Science, Ain Shams University, Cairo, Egypt; 6 Biological Control Research Department, Plant Protection Research Institute, Agricultural Research Center, Giza, Egypt; University of Oxford, UNITED KINGDOM OF GREAT BRITAIN AND NORTHERN IRELAND

## Abstract

*Pseudomonas aeruginosa*, recognized by the World Health Organization as a critical priority pathogen, exhibits significant genomic plasticity and a high potential for developing resistance to multiple antimicrobials. This study provides comprehensive genomic insights into colistin-resistant *P. aeruginosa* isolates obtained from cancer patients. Phenotypic assays were conducted to evaluate antibiotic susceptibility, biofilm formation, efflux pump activity, swarming motility, and pigment production. Whole genome sequencing of the collected isolates was performed using Oxford-Nanopore technology to examine sequence types, resistome profiles, virulence-associated genes, and mobile genetic elements. Our findings reveled that **o**ut of 52 isolates, 10 (19.2%) were resistant to colistin. Ceftolozane/tazobactam demonstrated full efficacy against 60% of colistin resistant *P. aeruginosa* isolates. Within this colistin resistant subset, high-risk clones ST308 and ST773 emerged as dominant, both harboring *bla*_*NDM-1*_ and exhibiting extensive resistance profiles, including resistance to colistin and, in some cases, ceftolozane/tazobactam. The first detection of ST1143 and ST1693 in Egypt carrying *bla*_*OXA-1028*_ and *bla*_*OXA-904*_, respectively was documented, neither of which had been previously reported in the country. The accessory genome, accounting for up to 34.6% of the total genome, highlights the remarkable genomic plasticity of *P. aeruginosa*, and its capacity for horizontal acquisition of resistance and virulence genes via mobile genetic elements, such as integrative and conjugative elements (ICEs). Virulome analysis revealed the presence of the *exoU* gene in high-risk clones, a marker closely linked to hypervirulence in infection models, whereas other sequence types were associated with less virulent factors, such as *exoS*. Despite phenotypic variability in biofilm formation, pigment production, and motility, the underlying genetic determinants of these traits were highly conserved. Mutational analysis revealed mutations in the regulatory system PhoPQ as the primary mechanism of colistin resistance, with no *mcr* genes detected. **In conclusion,** the substantial genomic plasticity of *P. aeruginosa*, reflected by an extensive accessory genome facilitates horizontal gene transfer (HGT), and significantly influences antimicrobial resistance and virulence. Colistin resistance was predominantly mediated by chromosomal mutations. Virulome and resistome analyses underscores the high pathogenicity and resistance potential of high-risk clones ST773 and ST308. The detection of horizontally acquired elements, such as integrative and conjugative elements (ICEs) carrying resistance genes such as *bla*_*NDM-1*_, underscores their role in disseminating resistance determinants. These findings emphasize the need urgent for targeted antimicrobial stewardship and surveillance strategies within Egyptian healthcare settings.

## 1. Introduction

The increasing antimicrobial resistance among healthcare-associated bacterial pathogens continues to restrict effective treatment options. Despite significant improvements in limiting the burden of health care-associated infections, outbreaks of multidrug-resistant (MDR) bacteria in healthcare settings continue to pose challenge in the treatment of vulnerable patient populations in hospitals [1]. *P. aeruginosa* has already been recognized as a key nosocomial pathogen, contributing significantly to mortality in hospitals worldwide [2]. It frequently evades the most widely used antimicrobial therapies through the acquisition and/or development of various resistance mechanisms [3]. Due to its high genomic plasticity and its potential resistance to a wide range of antimicrobials, *P. aeruginosa* has been designated as a serious threat by the Centers for Disease Control and Prevention and is considered a critical priority pathogen by the World Health Organization [4–7].

Of particular concern are carbapenem-resistant *P. aeruginosa* strains, many of which are simultaneously multidrug-resistant (MDR), extensively drug-resistant (XDR) or pan drug-resistant (PDR) [3,8]. Treating the infections caused by these problematic pathogens represents a significant challenge for clinicians, and may require the use of next-generation antibiotics, such as ceftolozane/tazobactam, and ceftazidime/avibactam [9–11]. Consequently, the treatment of carbapenem-resistant *P. aeruginosa* MDR/XDR isolates has led to a renewed on older antimicrobial agents, such as colistin, which is now regarded as a last-line therapy for infections caused by XDR non-fermentative Gram-negative bacteria [12,13].

However, the increased utilization of colistin, despite its potential antimicrobial activity, has contributed to the emergence of bacterial strains with reduced susceptibility to colistin worldwide [14]. Given the potential for horizontal gene transfer via conjugative plasmids and mobile genetic elements, as well as vertical transfer through chromosomal mutations, the rising incidence of colistin-resistant Gram-negative bacterial isolates poses a significant global public health threat, particularly among XDR or PDR clinical isolates [15].

Whole genome sequencing (WGS) - which includes sequencing of both the chromosome and mobile genetic elements) - offers the highest resolution for discriminating even closely related bacterial lineages. It has revolutionized outbreak analysis in hospital settings by offering extensive data on resistant problematic pathogens. Data generated from antimicrobial resistance (AMR) surveillance using WGS offer critical insights for developing rapid diagnostic tools, facilitating faster and more thorough AMR profiling, and complementing conventional phenotypic methods, ultimately improving the patients outcomes [16].

The increasing reports of MDR, XDR, and PDR *P. aeruginosa* in critically ill hospitalized patients pose a significant public health challenge within Egyptian health care facilities. This trend contributes to delays in initiating appropriate antimicrobial therapy, reduced treatment success, and elevated mortality rates among vulnerable patient populations [17]. In response to these challenges, the present study aimed to provide a comprehensive genomic analysis of colistin resistant *P. aeruginosa* isolates collected from a tertiary oncology hospital in Egypt, offering insights into the dynamic and diverse nature of their genomes and the implications for the antimicrobial resistance profiles in *P. aeruginosa* clinical isolates.

## 2. Materials and methods

### 2.1. *P. aeruginosa* clinical isolates

Between January 2021 and December 2022, a total of fifty-two *P. aeruginosa* clinical isolates were recovered from hospitalized cancer patients with bacteremia at the National Cancer Institute (NCI), Cairo, Egypt. The underlying malignancies included solid tumors (SOT) in 62% of cases and hematological malignancy (HM) in 38%.

These isolates were detected using the BACTEC automatic blood culture detection system (Becton Dickinson, Sparks, MD, USA) in the Microbiology Laboratory, Department of Clinical and Chemical Pathology, NCI. Positive blood culture bottles were processed using standard microbiological techniques, including cultivation on cetrimide agar (Himedia, India), and oxidase test (Himedia, India). Further identification was carried out using the BD PHOENIX Automated Microbiology System (Becton-Dickinson Diagnostic Systems, Sparks, MD, USA) [18]. For long term storage, isolates were suspended in 20% glycerol broth and preserved at -80 °C. *E. coli* ATCC 25922 was used as the quality control strain for identification procedures.

### 2.2. Antibiotic susceptibility testing

The Kirby-Bauer disc diffusion method was used to assess the susceptibility of *P. aeruginosa* clinical isolates to 10 antimicrobial agents (piperacillin/tazobactam (P/T), ceftazidime (CAZ); cefepime (FEP), aztreonam (ATM), meropenem (MEM), imipenem (IMP), levofloxacin (LEV), ciprofloxacin (CIP), amikacin (AMK), and gentamicin (GEN)). For ceftolozane/tazobactam (C/T), the minimum inhibitory concentration (MIC) was determined using E-test strips (Liofilchem, Roseto degli Abruzzi, Italy). The MIC for colistin (CT) was determined by broth microdilution assay [19]. The results were interpreted according to the guidelines of the European Committee on Antimicrobial Susceptibility Testing. As the EUCAST 2022 guidelines do not provide specific breakpoints for gentamicin against *P. aeruginosa,* the disc content and the interpretive criteria for Enterobacterales were applied instead [19].

*P. aeruginosa* isolates were classified according to the criteria proposed by Magiorakos et al. (2011) and Coyne (2022) into the following categories: i) Multidrug resistant (MDR): resistance to at least one drug in three categories of antimicrobials; ii) Extensively drug resistant (XDR): resistance to all but two or fewer categories of antimicrobials; iii) Difficult to treat resistance (DTR): non-susceptibility to all of the following: piperacillin-tazobactam, ceftazidime, levofloxacin, cefepime, aztreonam, meropenem, imipenem-cilastatin, ciprofloxacin; and iv) Pan drug resistant (PDR): resistance to all antimicrobials agents [3,20].

### 2.3. Phenotypic detection of virulence traits

Biofilm formation was assessed and interpreted following the method described by Sherif et al. (2021). The ethidium bromide (EtBr) cartwheel test, as outlined by Kothari et al. (2022), was used to evaluate efflux pump activity [21,22]. Pigment production and swarming motility were tested according to the protocols described by Alonso et al. (2020) and Ha et al. (2014), respectively [23,24].

### 2.4. *In-vivo* pathogenic potential assay

The non-mammalian host *Galleria mellonella* larvae was used as an infection model to assess strain pathogenicity, minimizing interference from patients-associated factors. Groups of 10 larvae were injected with 10 µL of bacterial suspension (10^4^ CFU/mL), delivering an approximate dose of 10^2^ CFU/larva. Injections were performed using a Hamilton syringe in the last left pro-leg of the larvae’s abdomen. An additional control group of 10 larvae was injected with 10 µL of phosphate-buffered saline (PBS) and served as the negative control [25,26].

### 2.5. Genomic analysis of MDR/XDR and colistin resistant *P. aruginosa* isolates

Ten MDR/XDR and colistin resistant *P. aeruginosa* isolates were selected for WGS analysis.

#### 2.5.1. Genomic DNA extraction and assessment.

A single colony from each bacterial isolate was aseptically transferred into 2 mL of Luria-Bertani (LB) medium and incubated overnight at 37^o^C. Bacterial DNA was extracted using the PureLink Microbiome DNA Purification Kit (Thermo Fisher Scientific, USA) following the manufacturer’s protocol then stored at 20°C until used for library preparation. DNA purity was verified using a Nanodrop One spectrophotometer, and DNA concentration was measured using the Qubit dsDNA HS kit with a Qubit 4.0 fluorimeter [27].

#### 2.5.2. DNA library construction and whole genome sequencing.

Library preparation was performed using Nanopore Rapid Barcoding Kit (SQK-RBK110.96) (Oxford Nanopore Technologies, UK) following the manufacturer instructions. Briefly, the barcoded tags were attached to the DNA ends. Sequencing adapters supplied in the kit were subsequently attached to the DNA ends. Finally, the MinION Flow Cell (R10.4.1) was primed, and the prepared DNA library was loaded into the flow cell. Sequencing was carried out using MinION Mk1C device (Oxford Nanopore Technologies, UK) [28].

#### 2.5.3. Bioinformatic analysis.

Raw FASTQ reads subjected to quality assessment using Nanoplot tool (http://nanoplot.bioinf.be/) [29]. Reads with low quality or insufficient length were excluded using NanoFilt tool (https://github.com/wdecoster/nanofilt) [30] applying a minimum quality threshold of “8”. Taxonomic classification of the samples was performed using Kraken2 tool, along with the Kraken (v. 20210127) database (https://benlangmead.github.io/aws-indexes/k2) [31].

The resultant filtered reads then used for *de novo* assembly and polishing employing Flye (v. 2.9.1-b1780) and Medaka (v. 1.7.2) tools (https://github.com/nanoporetech/medaka), respectively [32]. Medaka was used to correct residual base-calling errors, as it has been shown to produce more accurate results than Nanopolish. Medaka uses a neural network and comes with trained models that correspond to specific combinations of Oxford Nanopore Technologies (ONT) chemistry and basecaller combinations [33].

Following assembly, the genome quality was evaluated using Quast (v. 5.2.0) (https://quast.sourceforge.net/) [34]. Genome annotation was performed with Prokka (v.1.14.5) (https://usegalaxy.eu/?tool_id=prokka) [35], and the resulting annotation files were incorporated into genome quality assessment. Further validation was conducted using CLUSTALO alignment tool (https://www.genome.jp/tools-bin/clustalw) by aligning the the assembled genomes and protein sequences of the isolates to the reference genome *P. aeruginosa* strain PAO1. This ensured the consistency of base calling and protein sequences accuracy across the genome and confirmed that the alignment scores met the required threshold [36].

The Multi-Locus Sequence Typing (MLST) of the isolates was determined from the whole genome sequences. Sequence types were obtained from assemblies using Pathogenwatch (https://pathogen.watch/) and the *P. aeruginosa* database available at PubMLST [37]. For comparative phylogenetic analysis, *P. aeruginosa* genome sequences with full-length chromosome assemblies generated using Oxford Nanopore long-read sequencing technology were selected from the NCBI database. Multiple sequence alignment (MSA) and phylogenetic construction were performed using Parsnp (v 2.0) (https://github.com/marbl/parsnp), with default parameters used for alignment [38]. Upon completion of the alignment, a maximum likelihood phylogenetic tree was constructed. The resulting tree was visualized and further annotated using iTOL (https://itol.embl.de/) to highlight phylogenetic clades and relationship among samples within the dataset [39].

Antimicrobial resistance determinants (AMR) were identified using the Comprehensive Antibiotic Resistance Database (CARD) (https://card.mcmaster.ca/) and Amrfinderplus (https://github.com/ncbi/amr) with MUT parameters [40,41]. Mutations in the colistin resistance-determining regions were manually inspected using the Integrative Genomics Viewer (IGV) (https://igv.org/app/), allowing verification of variant calls across multiple reads and the detection of any anomalies indicative of potential sequencing errors. Read depth of these genes were analyzed by extracting aligned reads from BAM files using samtools (https://www.htslib.org/doc/samtools.html). Mutations were considered reliable if covered by a minimum average depth of >40× for the gene sequence. Possible mutations that were not curated in the AMRfinder or CARD database were assessed using Protein Variation Effect Analyzer tool PROVEAN (https://www.jcvi.org/research/provean) (version 1.1) in order to predict whether the detected mutation had an impact on the biological function of the protein or not with a default score threshold set at -2.5 [42].

Virulence factors (VFs) were identified using the NCBI, VFDB (http://www.mgc.ac.cn/VFs/main). Plasmid replicons in the plasmid sequences were inspected using Abricate (https://github.com/tseemann/abricate) and PlasmidFinder (https://bio.tools/PlasmidFinder). Insertion sequences were identified using blastn (https://www.ncbi.nlm.nih.gov/geo/query/blast.html) [43], utilizing the ISFinder sequences database [40,41,44]. Integrative and conjugative elements (ICE) were detected using ICEfinder web-based tool (https://bioinfo-mml.sjtu.edu.cn/ICEfinder/ICEfinder.html).

Based on a total of 10,813 genes from 10 isolates, pangenome analysis was performed using the Roary pipeline (https://bio.tools/roary), covering 9,547,506 bp to form the pangenome [45]. Consequently, the *P. aeruginosa* pangenome was categorized into core and accessory genomes. The accessory genome was further subdivided into two gene sets: strain-specific or shell genes, which are present in only a single isolate, and cloud genes, which are found in multiple but not all isolates. The pangenome sequence was annotated using Bakta (https://bakta.computational.bio/) annotation tools.

Circular Genomes were generated using Proksee tool (https://proksee.ca/) annotated with antimicrobial resistance (AMR) genes and mobile genetic elements (MGE).

### 2.6. Ethics approval and consent to participate

The study received approval from the Safe Handling and Disposal of Chemicals and Biologicals committee at the Faculty of Pharmacy, Cairo University, Egypt (ML2836). The bacterial isolates were obtained from the Microbiology Lab as part of routine diagnostic investigations for patient care. Since there was no direct or indirect contact with patients and no identifiable patient data was used, obtaining informed consent was not necessary. All experiments were conducted in strict accordance with relevant ethical guidelines and regulations.

## 3. Results

Most of the isolates (n = 35, 67.31%) were classified as MDR, while 16 isolates (30.77%) were XDR, and only one isolate (1.92%) displayed a PDR phenotype. Additionally, 19 isolates (36.5%) were identified as DTR. The lowest resistance rates were observed with colistin (19.2%), aztreonam (48.1%) and ceftolozane/tazobactam (56.8%). Ten isolates were non susceptible to colistin and were selected for the study.

### 3.1. Genome assembly and MLST results

The raw sequencing reads for the 10 *P. aeruginosa* isolates have been deposited in the NCBI Sequence Read Archive (SRA) under BioProject accession number PRJNA1131125. The corresponding assembled genome sequences, generated using Flye and polished with Medaka, have been submitted to the NCBI GenBank database under the same BioProject accession number. S1 Table lists the deposition numbers and the complete features of the genomes. Sequence types were determined using PubMLST based on the assembled genome data. Six distinct sequence types (ST308, ST664, ST773, ST381, ST1143, and ST1693) were identified and assigned to isolates P39, P46, P48, P55, P32, P1, P36, and P38, respectively. Isolates P39, P46, and P48 have the same ST308 which is known as an MDR- high risk clone. For isolates P19 and P24, sequence types could not be identified through PubMLST; therefore, Pathogenwatch was used and revealed a single locus variant in *guaA*, with closest match to ST773.

### 3.2. Identification of the AMR resistance mechanisms revealed by WGS analysis

The antibiotic susceptibility phenotype results of the ten isolates revealed that all were non-susceptible to ceftazidime, and cefepime. Susceptibility to levofloxacin, amikacin, and gentamicin was observed in only one (10%) isolate for each. Piperacillin-tazobactam and ciprofloxacin susceptibility were retained by only two isolates. Susceptibility to carbapenems (imipenem and/or meropenem) and aztreonam was observed in four (40%) isolates for each. Meanwhile ceftolozane-tazobactam showed susceptibility in six (60%) isolates, which was the highest among the ten colistin resistant isolates.

**Fig 1:**
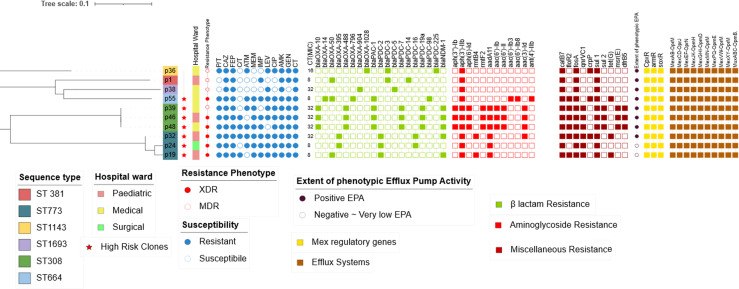
Overview of the antimicrobial susceptibility profiles and resistance determinants. The hospital ward, resistance profile, colistin MIC, and sequence types of isolates are depicted followed by resistance genes for different classes identified by the CARD system and the AMRfinder. Each class is identified by the color code shown below the figure.

The WGS analysis revealed the presence of a wide repertoire of resistance elements, with a total of 43 resistance genes identified using the NCBI tool (AMRfinder) and the CARD database. These genes were distributed among the isolates in combinations ranging from 7 to 24 genes per isolate. Isolates P39, P46, and P48, which belong to the high-risk clone ST308 showed the highest number of co-existing resistance genes, with 24 genes per isolate. The antimicrobial susceptibility profile, resistance determinants, and sequence types of the isolates are depicted in Fig 1.

**Fig 2:**
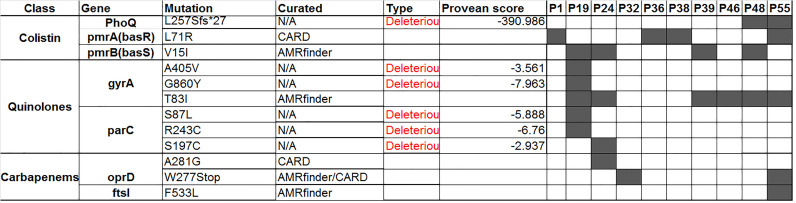
Overview of some gene mutations (Deleterious) in the sequenced *P. aeruginosa* clinical isolates.

Furthermore, genome analysis revealed the presence of gene clusters responsible for efflux pump-mediated resistance. More than 30 efflux genes were shared among all isolates, encoding membrane fusion proteins (MFPs), resistance nodulation division proteins (RNDs), and outer membrane proteins (OMPs) that constitute efflux pump systems. These gene clusters indicate the presence of ten chromosomally encoded multidrug resistance efflux pumps including MexAB-OprM, MexCD-OprJ, MexEF-OprN, MexGHI-OpmD, MexJK-OpmH, MexMN-OprM, MexPQ-OpmE, MexXY-OprM, MexVW-OprM, and MuxABC-OpmB. All isolates harbored the Mex-regulatory genes *armR*, *cpxR*, and *soxR*. These efflux systems confer resistance to a wide range of antibiotic classes. Additionally, mutation analysis revealed deleterious mutations associated with antimicrobial resistance for different classes with Provean score less than -2.5 as seen in Fig 2.

#### 3.2.1. Colistin resistance.

**Fig 3:**
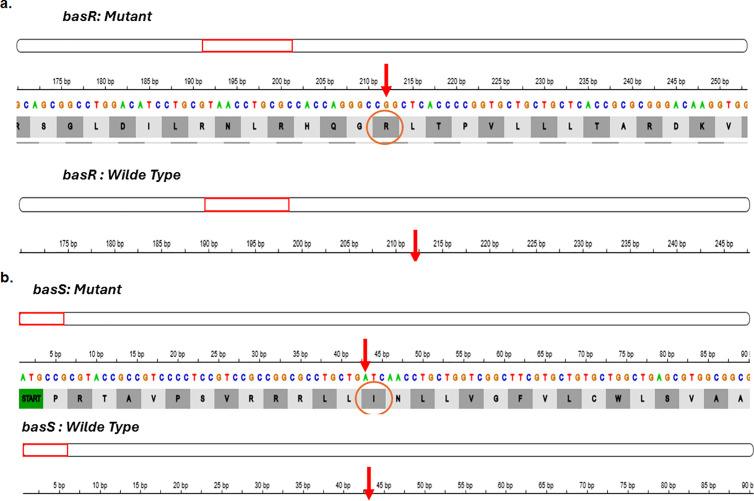
Mutation analysis for genes associated with colistin resistance. **a.** Mutation (L71R) in basR regulatory gene (also known as pmrA) detected in isolates P1, P36, P38 and P55. **b.** Mutation (V15I) in basS (also known as pmrB) detected in isolates P19, P24, P39, P48.

**Fig 4:**
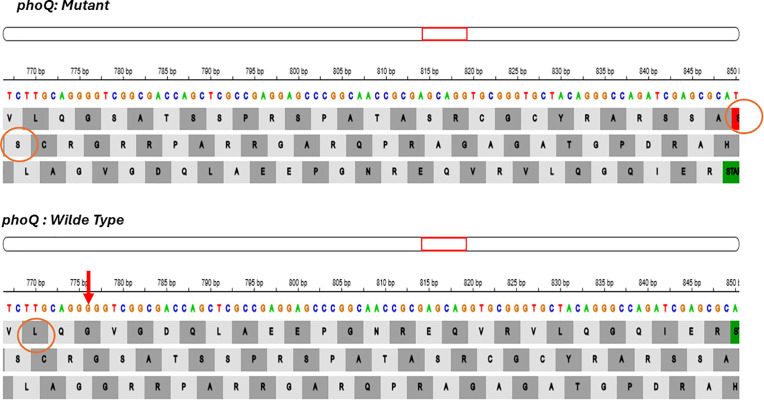
Mutations (L257Sfs*27 (776delG)) in phoQ gene in isolates P48 and P55.

Colistin resistance was chromosomally mediated by mutations in the basRS (pmrAB) and phoPQ regulatory systems. Mutation analysis for genes associated with colistin resistance revealed non-synonymous mutations in the two-component regulatory system basRS in isolates (P1, P19, P24, P36, P38, P39, P48, P55) (Fig 3). A mutation in *phoQ* gene, which is part of the phoPQ two-component system were detected in which a single nucleotide deletion at position 776 of the coding DNA sequence (c.776delG) was identified in isolates (P48, P55), resulting in a frameshift mutation. This mutation altered the original reading frame beginning at codon 257, where the wild-type leucine is replaced by a serine. The frameshift resulted in the incorporation of 26 aberrant amino acids followed by a premature stop codon, resulting in a truncated protein (p. L257Sfs*27) (Fig 4).

#### 3.2.2. B-lactams resistance.

Several variants of β-lactam hydrolyzing enzymes including *bla*_*OXA-50*_ family (*bla*_*OXA-50,*_ *bla*_*OXA-395,*_ *bla*_*OXA-488,*_ *bla*_*OXA-907,*_ and *bla*_*OXA-1028*_), *bla*_*OXA-1*_ family (*bla*_*OXA-10*_*, bla*_*OXA-14,*_ *bla*_*OXA-796*_), Pseudomonas-derived cephalosporinase (PDC) along with *bla*_*PAC-1*_ and *bla*_*NDM-1*_ all of which conferring resistance to β-lactam antibiotics were detected among our collection. All isolates exhibited a co-existence of at least one *bla*_*OXA*_ variant along with one *bla*_*PDC*_ variant. Co-existence of 6 β-lactamase genes (*bla*_*OXA-10,*_ *bla*_*OXA-488,*_ *bla*_*PAC-1,*_ *bla*_*PDC-7,*_ *bla*_*PDC-19a*_*,* and *bla*_*NDM-1*_) were observed in ST308. While co-existence of 5 β-lactamase (*bla*_*OXA-14*_*, bla*_*OXA-50*_*, bla*_*OXA-796*_*, bla*_*PAC-1*_*,* and *bla*_*PDC-98*_) genes were observed in P55 ST664.

Ceftolozane/tazobactam resistant isolate P19 showed co-existing B-lactamase genes *bla*_*OXA-50*_*, bla*_*PDC-2*_*, bla*_*NDM-1*_. Isolate P32 showed co-existing *bla*_*OXA-395*_*, bla*_*PDC-2*_*, bla*_*PDC-16*_*, bla*_*NDM-1*_ while P46 and P48 contained *bla*_*OXA-10*_*, bla*_*OXA-488,*_ *bla*_*PAC-1*_*, bla*_*PDC-7*_*, bla*_*PDC-19a*_*, bla*_*NDM-1*_. No specific mutation patterns were found among the C/T resistant isolates.

Aztreonam resistant isolate P24 harbored *bla*_*OXA-395*_*, bla*_*PDC-2*_*, bla*_*PDC-16*_, P32 showed the same genetic combination along with *bla*_*NDM-1*_. P36 harbored *bla*_*OXA-1028*_*, bla*_*PDC-3*_*,* and *bla*_*PDC-225.*_ P39 and P48 shared the same genetic combination in which *bla*_*OXA-10*_*, bla*_*OXA-488*_*, bla*_*PAC-1*_*, bla*_*PDC-5*_*,* and *bla*_*PDC-19a*_ and *bla*_*NDM-1*_. genes were detected in both isolates. P55 contained *bla*_*OXA-14,*_ *bla*_*OXA-50,*_ *bla*_*OXA-796,*_ *bla*_*PAC-1*_*,* and *bla*_*PDC-98*_.

**Fig 5:**
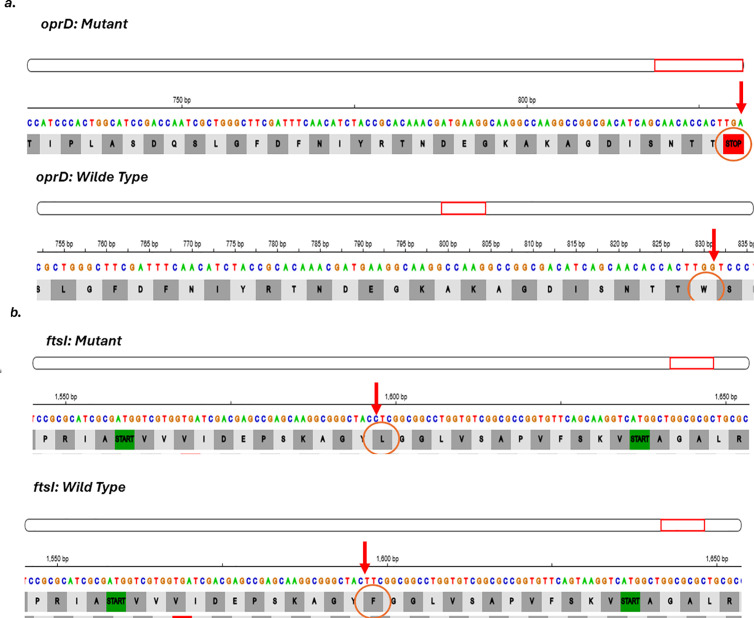
Point Mutations in oprD and ftsI Genes in *P. aeruginosa* isolates. **a.** Mutation (W277STOP) in oprD gene detected in isolates P32 and P55. **b.** Mutation (F533L) in ftsI gene detected in isolate P55.

The endemic *bla*_*NDM-1*_ was detected in five carbapenem resistant isolates belonging to ST773 and ST308. Six variants of *bla*_*OXA*_ genes and five *bla*_*PDC*_ variants were distributed among the carbapenem resistant isolates as shown in Fig 1. A deletion mutation in the outer membrane porin *oprD* was detected in P32 and P55. Additionally, one amino acid change in the *ftsI* gene encodes PBP3 (penicillin binding protien3) which was detected in P55 (Fig 5).

#### 3.2.3. Other antibiotic resistance genes.

Twelve aminoglycoside modifying enzymes were distributed across all isolates (Fig 1). Among these, the aminoglycoside phosphotransferase *aph(3’)-IIb* was detected in all isolates. The aminoglycoside nucleotidyltransferase *aadA11*was present only in isolates belonging to ST773 and ST308 (60%), and the 16S-rRNA methyltransferase *rmtF2* was detected in isolates belonging to ST308 (30%). Additionally, rmtB*4* was detected in P19 and P32 belonging to ST773 (20%). No non-synonymous mutation associated with aminoglycosides resistance was detected.

Quinolone resistance was attributed to four resistance genes, including the novel fluoroquinolone resistance gene *crpP* (found in P32, P36, and P55) and the integron-mediated quinolone resistance protein *qnrVC1*which was detected in six isolates as shown in Fig 1. Several non-synonymous mutations in the *gyrA*, *parC* were also identified, contributing to the quinolone resistance (Fig 2).

The *fosA* gene, encoding the fosfomycin resistance glutathione transferase, and the *catB7* gene, a chromosome-encoded variant of the chloramphenicol O-acetyltransferase *cat* gene were detected in all isolates. Additionally, the chloramphenicol efflux MFS transporter *floR2* gene was found in 60% of the isolates (Fig 1). Isolates P19 and P55 were the only two exhibiting mutations in *glpT* gene.

The *sul1*, sulfonamide resistant dihydropteroate synthase gene was detected only in the high-risk clones P19, P24, P39, P46, P48, P55 (70%), while plasmid linked *sul2* was detected in all isolates belonging to high-risk clone ST308. Other detected resistance genes include trimethoprim-resistant dihydrofolate reductase *dfrB5*gene and the ABC-F type ribosomal protection protein Msr(E) which confer resistance to macrolides; both were detected in ST308. The tetracycline efflux MFS transporter Tet(G) gene was detected in P19, P32, P55 (30%).

### 3.3. Detection of virulence factors phenotypes

The ten isolates selected for WGS analysis were assessed for the biofilm formation ability, the EPA, and the *in-vivo* pathogenic potential assay. The results revealed that all the isolates were biofilm producers. Among them, three displayed strong biofilm formation ability (P19, P24, and P32), six exhibited moderate biofilm formation ability (P36, P38, P39, P46, P48, and P55), and only one showed a weak biofilm formation ability (P1). The results of EPA test showed that two *P. aeruginosa* isolates (P19 & P24) fluoresced at EtBr concentration of (0.5 mg/L), therefore, they were considered negative or have very low EPA. The remaining isolates (n = 8) that fluoresced at higher EtBr concentration were considered positive EPA.

Three out of the ten isolates were pyocyanin producers (P1, P32, and P36) while the rest were pyoverdine producers. Swarming motility was observed among eight isolates out of the ten in which P1 and P19 were the only isolates with no swarming phenotype.

**Fig 6:**
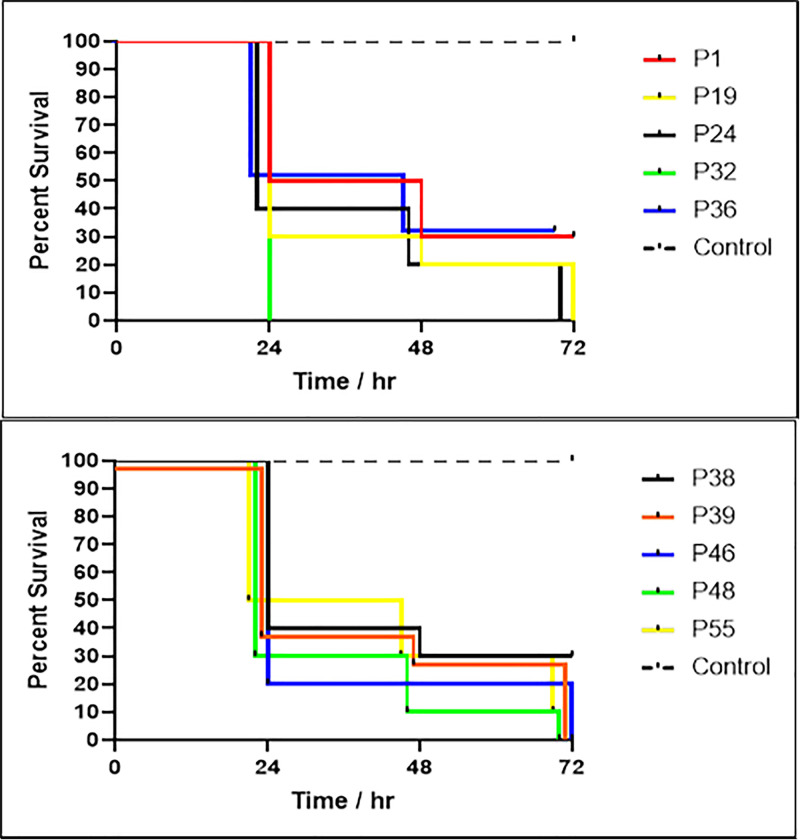
Kaplan-Meier survival curve of *G. mellonella* infected with different *P. aeruginosa* isolates to detect strain pathogenicity. Time readings were after 24, 48, and 72 hrs sharp, the nudge seen in the graph is just for avoiding curve overlapping.

The *in- vivo* pathogenic potential of each isolate was assessed based on the percentage of death among the infected *G. melonella* larvae and the results are shown in Fig 6.

### 3.4. Detection of genotypic virulence

**Fig 7:**
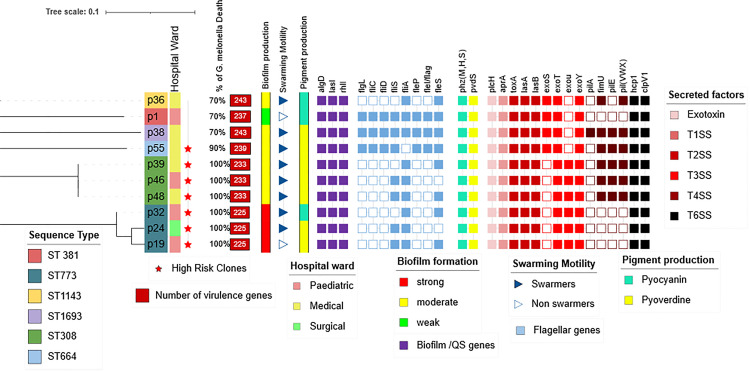
Overview of the phenotypic and genotypic virulence traits of the ten *P. aeruginosa* isolates along with the results of the in-vivo pathogenic potential test.

The NCBI VF database was used to detect the presence of genes accountable for virulence phenotypes and severity of infections. A vast set of genes encoding VF ranging from 225 to 243 genes per isolate were identified. Results from the analysis of the VF genes are summarized in Fig 7 in terms of numbers of virulence genes and presence or absence of some selected genes related to each factor.

All isolates harbored the alginate production gene set (*alg*) and QS genes (*rhlI* & *lasI)*. Type II secretion system genes *lasA* and *lasB* (protease and elastase encoding genes) were detected among all isolates along with the 11 *xcpQ ~ Z*. Additionally, the *plcH* (phospholipase) gene, *toxA* (exotoxin A) gene, and *aprA* (alkaline protease) gene were also detected among all isolates. Furthermore, the four T3SS effectors were detected in different patterns, in which *exoT* & *exoY* were detected among all isolates. On the other hand, the co-occurrence of *exoS* & *exoU* was found to be mutually exclusive. All type T4 pili genes were detected among all isolates except for the major pilin *PilA*, and minor pilins *fimU* and *pilVWXE* that their presence was varied. The H1-T6SS Hcp1and ClpV1proteinencoding genes were present in all isolates, no H2-H3 T6SS genes or effectors were detected. A total of 46 genes related to structure and assembly of flagella were distributed among the isolates in which most of the genes were conserved among all isolates except for *flgL, fliC, fliD, fleI/flag, fliS, fleP,* and *fliA*. The *phzM*, *phzS*, and *phzH* genes involved in the conversion of phenazine compounds to pyocyanin were detected among all isolates along with the *pvdS* σ factor required for pyoverdine synthesis.

### 3.5. Pangenome analysis and characterization of core and accessory genomes

**Fig 8:**
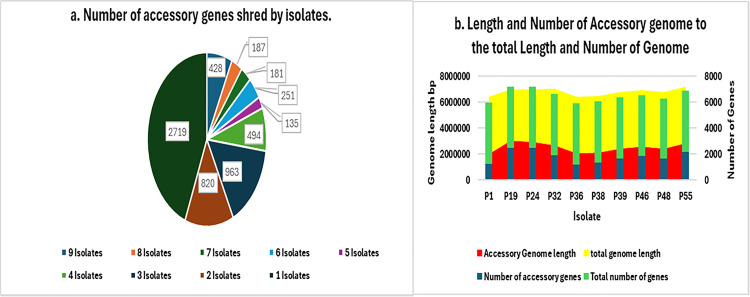
Accessory genome information. **a.** Number of accessory genes shared per isolate. **b.** Length and Number of Accessory genomes to the total Length and Number of Genome.

**Fig 9:**
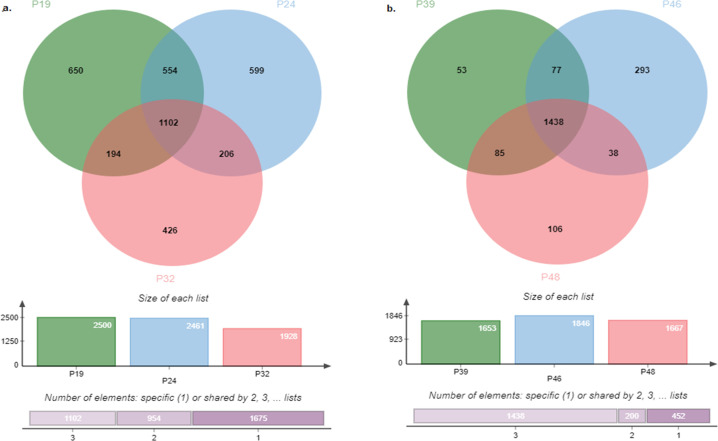
Venn diagram shows the frequency of co-existence of the accessory genome among the isolates of the same sequence type. **a.** ST773 (P19, P24, and P32). **b.** ST308 (P39, P46, and P48).

Pangenome analysis revealed the presence of 4634 (~42.86%) core genes and 6179 (~57.14%) accessory genes. The accessory genes consisted of 3460 shell genes which represent ~32% of the total genome and 2719 cloud genes which represent ~25.14% of the total. Isolates P19 and P24 that belong to the ST773 harbored the highest number of accessory genes (n = 2500 and 2461 respectively) followed by P55 (n = 2176). Isolate P1 was the least accessory genome harboring isolate (n = 1234). The distribution of the cloud/ shell genes between isolates, along with the length and number of accessory genes (cloud and shell) compared to total genes per isolate are represented in Fig 8. Results also revealed a co-existence of 1102 accessory genes in isolates P19, P24, and P32 (ST773) & 1438 accessory genes in isolates P39, P46, and P48 (ST308) (Fig 9).

### 3.6. Analysis of the horizontal gene transfer regions and mobile genetic elements

**Fig 10:**
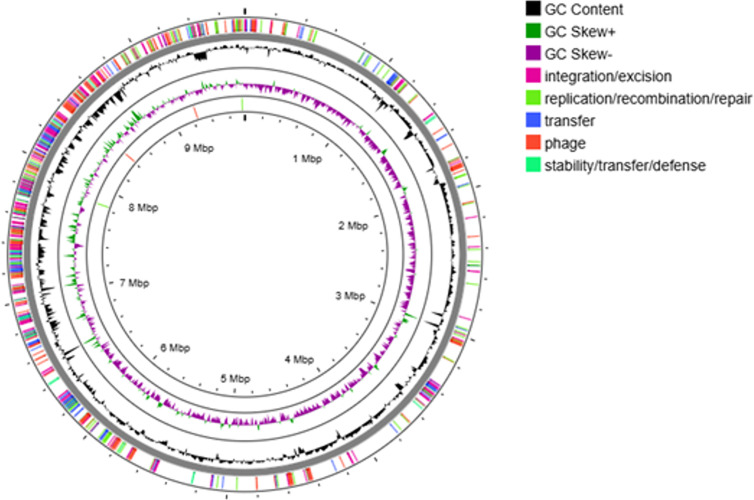
Circular representation of the pangenome of the ten sequenced *P. aeruginosa* isolates.

**Fig 11:**
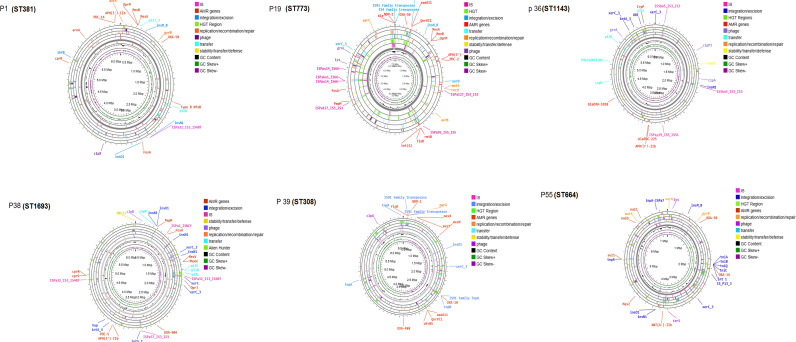
Circular genome of P1 (ST 381), P19 (ST773), P36 (ST1143), P38 (ST1693), P39 (ST308), and P55 (ST664) including tracks representing different elements of the accessory genome. The drawing also shows some of the important detected AMR genes with their surrounding environment.

The accessory genome of *P. aeruginosa* is predominantly composed of mobile genetic elements (MGEs) and regions associated with horizontal gene transfer (HGT). To further investigate these regions, the pangenome was analyzed using Mobile OG tool to identify the major components of the accessory genome. Fig 10 presents a circular representation of the pangenome assembly, consisting of a single representative nucleotide sequence from each of the clusters in the pan genome (core and accessory). This diagram highlights the identified MGE and the HGT regions. Additionally, each isolate was examined separately to characterize the MGE and HGT in each isolate using various tools, including Mobile OG, ISfinder, ICEfinder, and Alien Hunter (Table 1, Fig 11). The results revealed the presence of multiple integrative and conjugative elements (ICEs) carrying diverse sets of MGEs, antimicrobial resistance (AMR) genes, and virulence factors. Detailed information regarding these findings is provided in Table 2.

**Table 1:** The Number of Mobile Genetic Element (MGEs) genes present in different bacterial isolates categorized by their functional roles.

	Number of genes in Each Isolate									
**Type of the Mobile Genetic Element**	**P 1**	**P 19**	**P24**	**P32**	**P 36**	**P38**	**P39**	**P46**	**P48**	**P55**
Integration/excision	31	81	72	72	32	43	68	89	71	95
Replication/recombination/ repair	55	78	83	70	57	58	68	73	66	74
Phage	87	121	124	136	42	60	100	100	103	133
Stability/transfer/defense	4	19	17	19	12	20	18	25	17	24
Transfer	33	73	73	98	65	63	58	79	58	127
Insertion sequence	131	157	153	81	34	39	80	113	80	126

**Table 2:** List of the integrative & conjugative element (ICE) that carries important AMR and virulence genes within our *P. aeruginosa* collection as revealed by ICEfinder database.

Isolate	Number of ICE regions	Length/bp	Type	Proteins	Major AMR genes	Major Virulence genes	Region Annotation
P1							
P19	Region1	329304	Putative ICE with T4SS	327	*bla*_*OXA-50*_ family*, qnrVC1,*	Type IV SS (*virB10, pilZ*), Type VI SS (*vgrG, IcmF1,vgrG1a*), Type IIISS(*exoT*),*phzH*	P19- ICE region 1 Annotation
	Region2	113803	Putative ICE with T4SS	129	*rmtB4, floR2, tetR(G).*	Type II SS (*hicA*) toxin, STY4528 family pathogenicity island replication protein	P19- ICE region 2 Annotation
P24	Region 1	35594	Putative IME	29			P19-IME region 1 Annotation
	Region2	329280	Putative ICE with T4SS	322	*bla*_*OXA-50*_ family, *sul1*	Type IV SS (*virB10*), Type VI SS (*vgrG, hsiA1, vgrG1a*), catalase	P24- ICE region 2 annotation
	Region3	94546	Putative ICE with T4SS	112	*sul1*	STY4528 family pathogenicity island replication protein, Type II SS (*hicA*) toxin	P24 -ICE region 3 annotation
P32	Region 1	3109	Putative IME without identified DR	3			P32-ICE region 1 Annotation
	Region2	325939	Putative ICE with T4SS	289	*qnrVC1, sul1*	Type VI SS (*vgrG1a, hsiC1),* T3SS (*exoT*), Catalase	P32- ICE region 2 annotation
	Region3	116995	Putative ICE with T4SS	122	*bla*_*NDM-1*_ *, ble, floR2, tetR(G), rmtB*	STY4528 family pathogenicity island replication protein, Type II SS (*hicA*)toxin,	P32- ICE region 3 annotation
P36	Region1	89030	Putative ICE with T4SS	94	*tetR*	Type IVSS *pil (M, V2,U,R,P2,O2,X,L*), STY4528 family pathogenicity island replication protein	P36- ICE region 1 annotation
P38	Region1	108768	Putative ICE with T4SS	113	*tetR*	Type II SS *(hicA*)toxin, Type IV SS *pil(P2, N, O2, R, U, M*)	P38- ICE region 1 annotation
P39	Region1	248204	Putative ICE with T4SS	229	*tetR, bla*_*NDM-1*_*, floR, msr (E)*, *oprD* family porin	Type IVSS(*virB2*), type III SS (*exoY), algD*	P39- ICE region 1 annotation
	Region2	19611	Putative IME	17			P39- ICE region 2 Annotation
P46	Region1	200664	Putative ICE with T4SS	190	*bla*_*NDM-1*_*, floR, msr(E), tetR oprD* family porin,	Type III SS (*exoY),* algD	P46- ICE region 1 annotation
	Region2	151895	Putative ICE with T4SS	160		STY4528 family pathogenicity island replication protein, Type II SS (*hicA*),	P46- ICE region 2 annotation
P48	Region1	19611	Putative IME	18			P48-IME region 1 annotation
	Region2	338823	Putative ICE with T4SS	307	*bla*_*NDM-1*_, msr(E),floR, tetR	*algD*, type III SS (*exoY*)	P48- ICE region 2 annotation
P55	Region1	34345	Putative IME	30			P55-ICE region 1 annotation
	Region2	114162	Putative ICE with T4SS	120		Type IVSS *pil (T,M,X,R,P2,N),* STY4528 family pathogenicity island replication protein	P55- ICE region 2 annotation
	Region3	104778	Putative ICE with T4SS	116	*sul1, arsH, arsB, ant(4’)-Iib, floR2, tetR(G), tet(G)*	Type II SS (*hicA*) toxin	P55- ICE region 3 annotation
	Region4	359545	Putative ICE with T4SS	352	*ampR, bla*_*PDC-98,*_ *aph(3’)-Iib, oprD* family outer membrane porin		P55- ICE region 4 annotation

### 3.7. Phylogenetic analysis

**Fig 12:**
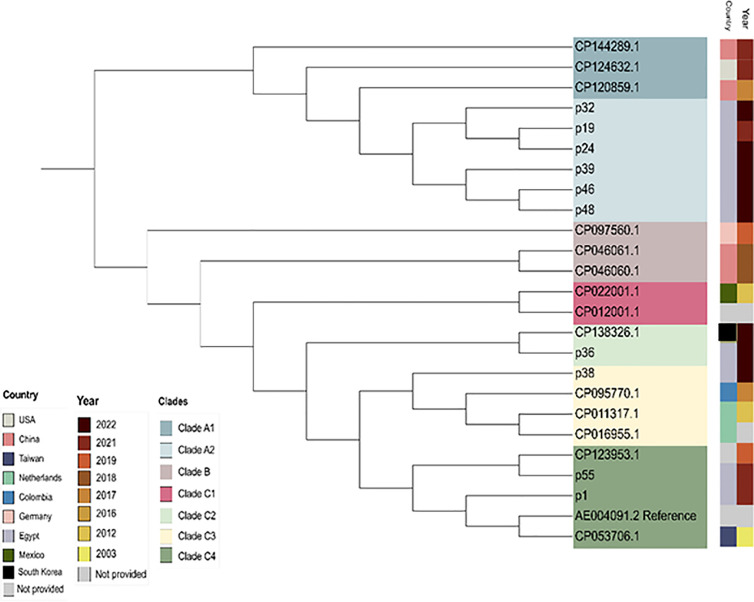
Phylogenetic tree of P. aeruginosa complete genomes sequenced in the current study compared to other strains retrieved from the NCBI according to the presence of complete chromosome sequences.

Phylogenetic analysis of the ten *P. aeruginosa* isolates from this study, along with 14 additional *P. aeruginosa* isolates retrieved from the NCBI (all possessing complete chromosome sequences generated using long-read Oxford Nanopore technology) revealed that our collection clustered into two main clades (Fig 12). Isolates P19, P24, P32, P39, P46, and P48, which belong to ST773 and ST308, were exclusively allocated to the subclade A2. This subclade exhibited genetic relatedness to subclade A1, which includes isolates recovered from China and USA. In contrast, isolates P1, P36, P38, and P55 were assigned to clade C. Among these, P36 grouped with subclade C2 along with an isolate (GenBank: CP138326.1) reported from South Korea in the same year. Isolate P38 was placed in the subclade C3, which contained three isolates from Colombia and Netherlands. Isolates P1 and P55 were assigned to subclade C4. Notably, P55 showed a close genetic relationship with an isolate from GenBank (CP123953.1); while isolate P1 was closely related to an isolate recovered from Taiwan.

## 4. Discussion

The widespread presence of multidrug-resistant (MDR) pathogens in Egyptian healthcare settings presents a significant barrier to effective infection control measures and surveillance, facilitating the spread of resistant strains. This study aimed to utilize whole-genome sequencing (WGS) to analyze colistin resistant *P.aeruginosa* clinical isolates recovered from hospitalized cancer patients. Our objectives were to characterize the population structure of these isolates, identify key virulence determinants, uncover antimicrobial resistance (AMR) mechanisms, and explore how different genomic lineages acquire these resistance determinants. In addition to the analysis of AMR mechanisms, pangenome analysis was performed to distinguish between core and accessory genes, providing valuable insights into the genetic diversity and plasticity of the isolates. By employing long-read Oxford Nanopore Technologies (ONT) sequencing, we were able to produce high-quality bacterial genome assemblies. The improvements in the yield and accuracy offered by long read sequencing allow for the generation of more complete and error-free genome sequences, which is crucial for accurately identifying resistance mechanisms and virulence factors in complex bacterial genomes [33].

Our findings revealed an alarming increase in the rate of extensively drug-resistant (XDR) and multidrug-resistant (MDR) phenotypes among *P. aeruginosa* clinical isolates particularly in response to antipseudomonal drugs and colistin, which remains the last resort therapeutic option in the Egyptian healthcare settings [15,46–48]. However, our study also identified a promising therapeutic option, with 60% of the colistin resistant *P. aeruginosa* isolates showing full (100%) susceptibility to ceftolozane/tazobactam. Previous studies have suggested that ceftalozane/tazobactam is an effective treatment for colistin resistant *P.aeruginosa* clinical isolates [49,50].

Invasive infections caused by pan-drug-resistant (PDR) *P.aeruginosa* in patients with oncohematologic diseases pose a significant therapeutic challenge. However, emerging treatment options and combined antibiotic therapies provide hope for improved patient outcomes. One promising approach is the use of high daily doses of amikacin monotherapy, adjusted to the minimum inhibitory concentration of the pathogen, in conjunction with continuous venovenous hemodiafiltration (CVVHDF) for treating severe sepsis caused by pan-resistant *P. aeruginosa.* Additionally, novel β-lactam/β-lactamase inhibitor combinations such as ceftolozane-tazobactam, ceftazidime-avibactam, imipenem-cilastatin-relebactam have shown a promising activity against difficult-to-treat *P. aeruginosa* strains [51].

The genomic configuration of the isolates exhibited significant variation in their resistome and virulome profiles, which were largely dependent on their sequence type (ST). Three multidrug-resistant (MDR) high-risk clones were identified, namely ST308, ST773, and ST664. Among these, ST773 and ST308 emerged as the dominant clones, with three isolates for each showing the highest prevalence. Additionally, isolates assigned to ST664 and ST381 were detected, both of which have been previously reported worldwide and in Egypt [46,52–55], and were associated with carbapenem resistance. Our findings, together with other studies, suggest that ST381 should be placed under heightened surveillance despite it was not being previously classified as an MDR-clone [56]. To the best of our knowledge, and according to pubMLST, both ST1693 and ST1143 had only been reported once, from China and Spain, respectively. Our study marks their second detection worldwide Notably, ST1143 and ST1693 exclusively harbored the *bla*_*OXA-50*_ family variant (*bla*_*OXA-1028*_) and (*bla*_*OXA-904*_), respectively. These variants had not been previously reported in Egypt. Moreover, ST308 and ST664 exclusively contained the *bla*_*PAC1*_ gene, which confers resistance to a wide range of β-lactam antibiotics, including ceftolozane/tazobactam (C/T). The presence of this gene within the chromosome of both sequence types has been previously reported [55,57]. Despite harboring the gene, only two isolates belonging to ST308 were resistant to C/T, indicating a potential variation in gene expression or additional resistance mechanisms that could influence the phenotypic outcome.

The three isolates assigned to ST308 harbored the *bla*_*NDM-1*_ gene, further emphasizing the clinical significance of this sequence type. ST308 has been identified as one of the top ten high risk clones globally strongly associated with multi-drug resistance, nosocomial infections and outbreaks [53,57–59]. Reports from Malaysia [60], Singapore [58], VietNam [59] and more recently Europe [57] have highlighted ST308 as a key reservoir for the spread of *bla*_*NDM-*1_. This study marks the first identification of three *bla*_*NDM-1*_ positive *P. aeruginosa* ST308 isolates in Egyptian hospitals, providing critical insight into the transmission dynamics of this high-risk clone within the local healthcare setting.

Similarly, ST773 has emerged as MDR high-risk clone worldwide [57,61–63] including more recently in Egypt [46,54]. In our study, two isolates belonging to ST773 also carried *bla*_*NDM-1*_ reinforcing the global spread of this resistance determinant. The *bla*_*NDM-1*_ gene was chromosomally integrated in both ST308 and ST773 clones, located within an integrative and conjugative element (ICE) that was present in all isolates belonging to ST308 and in isolate P32-ST773 (Table 2). This finding aligns with previous reports which have shown ICEs to play a pivotal role in the mobilization of *bla*_*NDM-1*_ across *P. aeruginosa* isolates [57,58,61].

The beta-lactamases genes *bla*_*OXA-10,*_ *bla*_*OXA-488,*_ *bla*_*PDC-7,*_ and *bla*_*PDC-19a*_ were exclusively detected in ST308. This sequence type also harbored the highest number of aminoglycoside resistance genes, including the *rmtF2* which confer pan aminoglycoside resistance and was absent in other isolates*,* Additionally, genes *sul2, msr(E), and drftB5* were also found in ST308. The genetic configuration of this clone was closely aligned with previously reported studies [57], indicating its persistent presence in the global spread of resistant *P. aeruginosa* clones.

In contrast, *bla*_*OXA-395,*_ *bla*_*PDC-2,*_ *bla*_*PDC-16*_ and *rmtB4 were* observed only *in* ST773. Interestingly, the resistome of ST773 in this study exhibited some differences when compared to the same sequence type in a recent Egyptian study [46]. Both ST308 and ST773 contained *aadA11, qnrVC1,* and *bla*_*NDM-1*_, contributing to their resistance profiles. Notably, resistance to ceftolozane/tazobactam (C/T) was observed only in ST773 and ST308, marking these as significant clinical threats.

In the *P. aeruginosa* genome, multiple drug efflux systems, predominantly from the RND and MFS families, contribute significantly to antimicrobial resistance [64]. In our study, we identified ten multidrug efflux tripartite pumps encoded in operons across all isolates, which surpasses the number detected in other Egyptian studies [65], underscoring the extensive resistance in our collection. Interestingly, although isolates P19 and P24 exhibited no or weak phenotypic efflux pump activity, the complete set of the ten efflux systems was present in their genomes. This finding aligns with previous studies which have demonstrated the existence of efflux pump genes even in the absence of noticeable phenotypic efflux activity [66].

In our study, colistin resistance was primarily attributed to chromosomal mutations in the two-component regulatory systems basSR (pmrAB) and PhoPQ. Specifically, we identified single amino acid change in *basR* and *basS* genes among 8 isolates. Additionally, a frameshift mutation was detected in *phoQ* genes among 2 isolates. Mutational resistance represent the most common causes of colistin resistance in *P. aeruginosa* clinical strains [67,68]. Although *mcr* genes have been detected in colistin resistant *P. aeruginosa in* Egypt [15,47], none of their variants were found in our collection. Notably, the *arnA* gene, which encodes an enzyme involved in lipid A modification, was retained in all isolates. Previous studies have shown that the *arnA* enzyme plays a crucial in the lipid A modification pathway, and its deletion can abolish polymyxin resistance in Gram-negative bacteria [69,70].

We assessed the pathogenicity of *P. aeruginosa* isolates’ using the *G. mellonella* model, a validated invertebrate infection model for studying virulence and pathogenicity [26,71]. Our findings showed that only the high-risk clones ST308 and ST773 harbored the *exoU* gene, while *exoS* was detected in the remaining sequence types. Notably, ST308 and ST773 induced 100% mortality in *G. mellonella* larvae, followed by ST664 which caused approximately 90% mortality. The other non-high-risk clones resulted in lower mortality rates (~70). This emphasizes the presence of a strong association between high-risk sequence types (ST308 & ST773) and the presence of the potent virulence *exoU* toxin, as opposed to the less virulence-conferring *exoS*, which has been reported previously [46,53,72]. Furthermore, in our collection the presence of *exoS,* and *exoU* was mutually exclusive, while *exoT,* and *exoY* were detected in all isolates, consistent from other studies [46,53,54].

Our virulome analysis also revealed the presence of other secreted factors, including *plcH*, *toxA*, *aprA, lasA* and *lasB* which encode phospholipase, exotoxin A, alkaline protease, protease and elastase, respectively, in all isolates. These factors are crucial for damaging host cells and enhancing pathogenicity [46,56,58,73,74] highlighting the hyper virulent nature of our collection. Our findings are in harmony with other studies that reported high prevalence of these virulence genes in clinical isolates [46,74–76]. In contrast, some studies have shown a lower prevalence of *toxA* [76,77]*,* which may be attributed the isolates’ ability to adapt well to conditions at the infection site and setting [76]. According to Bogiel et al, the presence of T4SS genes is not essential for the development of bacteremia following human colonization [78], which may explain the low prevalence of the *pilA* gene in our collection. This finding is consistent with other studies that reported low prevalence of *pilA* among blood stream isolates [78–80]. Notably, recent investigations have shown that *P. aeruginosa* employs H1-, H2-, and H3-T6SS mechanisms to deliver its toxins into both eukaryotic and prokaryotic cells [81]. In our study, we noted the presence of *hcp1*, and *clpV1*genes, which encode H1-T6SS components, in all of our isolates. However, we were unable to detect any of the H2 or H3- T6SS effectors. Both genes were identified in a recent study from Egypt [46].

Despite the fact that all the isolates were obtained from the bloodstream of different patients, they exhibited variability in phenotypic assays of biofilm formation, pigment production and swarming motility. Interestingly, we found that most of the genes responsible for the three phenotypes were highly conserved among the strains, despite their phenotypic variability. Furthermore, the absence or presence of certain genes associated with the swarming phenotype did not fully explain the phenotypic differences between the strains. This observation aligns with other studies that that also failed to demonstrate a clear correlation between the presence of specific genes and their corresponding phenotypic expression [66,76,82]. These findings suggest that the phenotypic variability is more likely attributable to regulatory changes, as suggested by Scheffle et al. [83].

*P. aeruginosa* has a genomic size ranging from 5.8 to 7.3 Mbp, with a core genome comprising over 4,000 genes, alongside a variable accessory gene pool [54]. The numbers of core and accessory genes can vary due to several factors, including the size of the genome population used for alignment, the type of the genome (incomplete draft or long read), and the diverse nature of the study [84,85]. In our pangenome analysis, the observed numbers of core and accessory genomes are consistent with those reported in other studies, including a recent study from Egypt [46,85].

Similar to many other bacteria, the accessory genomes of *P. aeruginosa* contains genes related to virulence and antibiotic resistance [85]. Previous studies have reported that the accessory genome of *P. aeruginosa* can account for up to 26% of the total genome, a figure they considered relatively high due to the use of draft genome in their study [85]. However, by employing long read sequencing to avoid the overestimating genome numbers as often seen with draft genomes, we found that the accessory genome makes up 34.6% of the total genome. This highlights the remarkable genomic plasticity of *P. aeruginosa* and its ability to incorporate a diverse array of foreign genes, which significantly contribute to its resistome and virulome.

The accessory genes in *P. aeruginosa* include phages, transposons, insertion sequences (IS) and integrative and conjugative elements (ICEs) [76,86]. These mobile genetic elements (MGEs) play a key role in the horizontal acquisition of antimicrobial resistance genes (ARGs), a mechanism that contributes significantly to the resistance in *P. aeruginosa* and and is a major concern in clinical settings [86]. In our study, we observed a notable association between different MGEs and the presence of specific resistance or virulence genes. For example, the *bla*_*NDM-1*_ gene was frequently found adjacent to IS91 family transposase genes, as seen in Fig 11. Other resistance genes, such as *sul1, floR, rmtB, tet(G*), *bla*_*NDM-1*_, *bla*_*PDC-98*_ and some *bla*_*OXA*_ variants, were mostly parts of ICEs, as detailed in Table 2. This finding is consistent with previous studies [62,86,87]. These ICES also carried various virulence factors, including the STY4528 family pathogenicity island replication protein, Notably, *exoY* and *algD* were detected in ST308, while *exoT* was present in *P. aeruginosa* isolates P19 & P32 ST773.

This study employed the long-read ONT to generate bacterial genome assemblies. While the results are promising, further improvements in sequencing yield and accuracy can be achieved by combining ONT long-reads with Illumina short-reads. Future research should focus on larger sample sizes and utilization of hybrid assembly techniques both (long-reads and short-reads) (hybrid assembly) to improve the depth and resolution of genome sequencing.

## 5. Conclusion

This study highlights the emergence of high-risk *P. aeruginosa* clones ST308 and ST773 in Egyptian hospitals, both of which carry the carbapenemase gene *bla*_*NDM-1*_ and exhibit extensive multidrug resistance profiles. These clones also harbor key virulence determinants, particularly the *exoU* genotype, which was associated with heightened pathogenicity in infection models. The large accessory genome (34.6%) observed highlights the pathogen’s genomic plasticity and its capacity for horizontal gene transfer, driving resistance and virulence. Notably, 60% of the colistin-non-susceptible *P. aeruginosa* isolates remained fully susceptible to ceftolozane/tazobactam, suggesting its potential as an effective therapeutic alternative. Despite phenotypic variability in traits such as biofilm formation, pigment production, and motility, the underlying genetic determinants governing these traits were largely conserved.

Pangenome analysis revealed a substantial accessory genome comprising 34.6% of the total genomic content, underscoring the remarkable genomic plasticity of *P. aeruginosa* and its capacity for horizontal gene acquisition. The presence of mobile genetic elements, including integrative and conjugative elements (ICEs) and insertion sequences (IS), played a significant role in the dissemination of resistance genes such as *blaNDM-1* and various virulence factors.

Overall, these findings provide a comprehensive genomic insight into the resistome and virulome of *P. aeruginosa* clinical isolates in Egypt and emphasize the urgent need for enhanced surveillance, molecular diagnostics, and targeted antimicrobial strategies within healthcare settings to mitigate the spread of these high-risk clones.

## Supporting information

S1 Table Genome annotation and accession numbers of the isolates. (DOCX)
