# Supplementary material for: The genomic configurations driving antimicrobial resistance and virulence in colistin resistant Pseudomonas aeruginosa from an Egyptian Tertiary Oncology Hospital
Source: PLOS Glob Public Health. 2025 Aug 5;5(8):e0004976. doi: 10.1371/journal.pgph.0004976 (PMC12324682; doi:10.1371/journal.pgph.0004976)
Supplement: S1 Table — (DOCX) [file pgph.0004976.s001.docx]

**S1 Table.** Genome annotation and accession numbers of the isolates.

| **Isolates** | **P1** | **P19** | **P24** | **P 32** | **P36** | **P38** | **P39** | **P46** | **P48** | **P55** |
| --- | --- | --- | --- | --- | --- | --- | --- | --- | --- | --- |
| **Accession Number** | SAMN42266025 | SAMN42266026 | SAMN42266027 | SAMN42266028 | SAMN42266029 | SAMN42266030 | SAMN42266031 | SAMN42266032 | SAMN42266033 | SAMN42266034 |
| **Match to the genus level** | 99.57 | 99.67 | 98.6 | 98.63 | 98.56 | 98.35 | 95.44 | 95.44 | 95.44 | 99.58 |
| **Number of Contigs** | 1 | 4 | 2 | 1 | 1 | 2 | 4 | 1 | 2 | 1 |
| **Largest contig** | 6421080 | 6950080 | 6941008 | 7056741 | 6424851 | 6444830 | 6795750 | 6949905 | 6797054 | 7209899 |
| **Total Length genome bp** | 6421080 | 7002210 | 6974984 | 7056741 | 6424851 | 6491576 | 6805831 | 6949905 | 6803993 | 7209899 |
| **Number of genes** | 5949 | 7226 | 7191 | 6651 | 5926 | 6067 | 6373 | 6558 | 6301 | 6896 |
| **CDS (Protein Coding Sequence)** | 5861 | 7136 | 7102 | 6563 | 5838 | 5977 | 6287 | 6472 | 6387 | 6810 |
| **GC (%)** | 66.39 | 65.79 | 65.81 | 65.89 | 66.36 | 66.29 | 66.14 | 66.04 | 66.14 | 65.73 |
| **N50** | 6421080 | 6950080 | 6941008 | 7056741 | 6424851 | 6444830 | 6795750 | 6949905 | 6797054 | 7209899 |
| **L50** | 1 | 1 | 1 | 1 | 1 | 1 | 1 | 1 | 1 | 1 |

* N50 is the shortest contig length that needs to be included for covering 50% of the genome.

* L50 is defined as count of smallest number of contigs whose length sum makes up half of genome size.
